# Involvement of Proteasome and Macrophages M2 in the Protection Afforded by Telmisartan against the Acute Myocardial Infarction in Zucker Diabetic Fatty Rats with Metabolic Syndrome

**DOI:** 10.1155/2014/972761

**Published:** 2014-07-08

**Authors:** C. Di Filippo, C. Rossi, B. Ferraro, R. Maisto, A. De Angelis, F. Ferraraccio, A. Rotondo, M. D'Amico

**Affiliations:** ^1^Department of Experimental Medicine, Section of Pharmacology “L. Donatelli”, Second University of Naples, 80138 Naples, Italy; ^2^Radiology, Radiotherapy and Nuclear Medicine Unit, Second University of Naples, 80138 Naples, Italy; ^3^Department of Clinical, Public and Preventive Medicine, Second University of Naples, 80138 Naples, Italy

## Abstract

This study investigated the involvement of proteasome and macrophages M2 in the protection afforded by telmisartan against the acute myocardial infarction in Zucker diabetic fatty (ZDF) rats with metabolic syndrome. ZDF rats were treated for three weeks with telmisartan at doses of 7 and 12 mg/kg/day. After treatment, rats were subjected to a 25 min occlusion of the left descending coronary artery followed by 2 h reperfusion (I/R). At the end of the I/R period, biochemical, immunohistochemical, and echocardiographic evaluations were done. Telmisartan treatment (7 mg/kg and 12 mg/kg) reduced the myocardial infarct size, the expression of proteasome subunits 20S and 26S, and the protein ubiquitin within the heart. The compound has led to an increased M2 macrophage phenotype within the cardiac specimens and a modification of the cardiac cytokine and chemokine profile. This was functionally translated in improved cardiac performance as evidenced by echography after 2 h reperfusion. 7 mg/kg/day telmisartan was sufficient to improve the left ventricular ejection fraction LVEF of the rat heart recorded after I/R (e.g., vehicle 38 ± 2.2%; telmisartan 54 ± 2.7%) and was sufficient to improve the diastolic function and the myocardial performance index up to values of 0.6 ± 0.01 measured after I/R.

## 1. Introduction

Our previous studies indicated a protective action of telmisartan, AT1 receptor blockers and peroxisome proliferator-activated receptors (PPAR) activator [1], against the acute myocardial infarction (AMI) occurring after ischemia/reperfusion in Zucker diabetic fatty (ZDF) rats with metabolic syndrome [2]. This study highlighted the fact that the protection was mainly exerted through a reduction of the proinflammatory circuits within the myocardium, including increased expression of adiponectin, reduced local NF-*κ*B and TNF-*α* production, and a downregulation of the TLR2 and TLR4 receptors within the cardiac tissue. However, having in mind the multifactorial aspects of the syndrome and the discovery of new factors within the scenario of the pathogenetic mechanisms underlying the cardiovascular risk from metabolic pathologies [2, 3] such as the nonlysosomal protein degradation system ubiquitin-proteasome (UPS), we aimed here to investigate whether the proteasome system is involved in the action of telmisartan against AMI in ZDF rats. This is in light of the prominent damaging role played by ubiquitin-proteasome system in the ischemic myocardium [4] and the correlation existing between telmisartan and ubiquitin-proteasome system in some pathologies [5]. Moreover, due to the fact that prosecution and/or resolution of inflammation is supported by different roles played by the two M1/M2 macrophage phenotypes, we also investigated whether the treatment of infarcted ZDF rats with telmisartan exerts any modification of the cardiac macrophage phenotypes.

## 2. Materials and Methods

Zucker diabetic fatty (ZDF) rats (320 ± 15 g, *n* = 38 total), purchased at 8 weeks of age from Charles River (Italy), were fed with a standard chow diet, tap water ad libitum, and were administered for the following three weeks with either vehicle (4 mL/kg/day of 1% methylcellulose) or telmisartan (7 and 12 mg/kg/day) by gastric gavage [2] (*n* = 10 per group). At the end of the treatment, 10 ZDF rats were anesthetized with urethane (1.2 g/kg intraperitoneally) and subjected to myocardial ischemia/reperfusion (I/R) injury for the evaluation of the tissue damage and for the echography as previously described with modifications [2].

### 2.1. Echocardiographic Evaluations

Echocardiography (Visualsonics Vevo 2100, Toronto) was performed before the starting of I/R procedure and after 2 h reperfusion (e.g., before the killing of the rats for morphologic evaluations). Images were obtained using a 10–14 MHz linear transducer for the measurement of morphometric parameters (left ventricular (LV) mass and relative wall thickness (RWT)) and systolic function (ejection fraction (EF), fractional shortening (FS), and velocity of circumferential fiber shortening (VCF)). Diastolic function was also evaluated by means of absolute LV isovolumetric relaxation time (IVRT), the ratio of maximal early diastolic peak velocity (*E*), and late peak velocity (*A*) of mitral flow (*E*/*A* ratio). The global index was quantified by the myocardial performance index (MPI). Echocardiographic parameters were measured in accordance with previous studies [6].

### 2.2. Measurement of Area at Risk (AR) and Infarct Size (IS)

Two hours after the reperfusion, the AR/left ventricle (LV), IS/AR, and IS/LV ratios were measured as previously described [2]. Selected experiments (*n* = 5 for each treatment, 10 total) were repeated, monitoring AR but omitting the staining procedures to determine IS. The AR was collected and half of each specimen was fixed by immersion in 10% buffered formalin and paraffin-embedded for immunohistochemistry. Sections were serially cut at 5 *μ*m, placed on lysine-coated slides, and stained with hematoxylin. The other half of each specimen was frozen at −80°C and used for biochemical assays described below.

### 2.3. Western Blot Analysis

Primary anti-20S proteasome *α*1/ *α*2/ *α*3/ *α*5/ *α*6/ *α*7 antibody (1 : 200, Santa Cruz Biotec, USA), anti-M2 mannose receptor (CD206) (1 : 400, Abcam, Cambridge, UK), anti-M1 Integrin Alpha X/CD11c (1 : 200, Bioss, USA), and primary antiubiquitin (1 : 200, Santa Cruz Biotec, USA) antibodies were used. Furthermore, the macrophages phenotypes were assayed with the aspecific MHCII antibody (1 : 200, Abcam, Cambridge, UK). Secondary antibodies used were donkey polyclonal to rabbit (1 : 1000, Abcam, Cambridge, UK) and goat antimouse (1 : 2000, Santa Cruz Biotec, USA).

### 2.4. Macrophages Extraction from Perinfarcted Myocardial Tissue

Macrophages were selectively extracted from perinfarcted myocardial tissue as previously described with modifications [7, 8]. Biochemical assays on cell homogenates for proteasome 20S determinations were performed as illustrated by a specific sodium dodecylsulfate activation kit (Boston Biochem, Cambridge, Massachusetts).

### 2.5. Cytokines Array

The levels of cytokines present in the cardiac tissue were determined using a commercial kit, Proteome Profiler Antibody Arrays (R&D Systems, Abingdon, UK), which allows us to simultaneously detect the relative levels of numerous cytokines and chemokines.

### 2.6. Immunohistochemistry

Sections from paraffin-embedded tissue were used according to previous published methods [2]. Sections were incubated with specific antibodies: anti-20S, 26S proteasome (1 : 10, Santa Cruz Biotec, USA) and ubiquitin (1 : 300, DAKO, Milan, Italy), anti-Mannose Receptor (CD206, 1 : 10, Abcam, Cambridge, UK), and anti-CD11c (1 : 100, Bioss, USA). Sections were washed with PBS and incubated with secondary antibodies. Specific labeling was detected with a biotin-conjugated goat antirabbit IgG and avidin-biotin peroxidase complex (DBA, Milan, Italy).

### 2.7. Statistical Analysis

The data obtained were analysed by ANOVA for normally distributed data and Kruskal-Wallis test for nonnormally distributed data. Bonferroni test was also used to make pairwise comparisons. *P* < 0.05 was considered significant and SPSS2 software was used.

## 3. Results

### 3.1. Effects of Telmisartan on the Myocardial Tissue Damage

Following the I/R procedure, the heart of the ZDF rats developed a myocardial injury characterized by an extension of infarct size of the 68% of the area at risk and by an extension of 36% of the infarct size/left ventricle. Area at risk was 61% of the left ventricle (Table 1). Approximately 30% (*P* < 0.05 versus vehicle) and 56% (*P* < 0.01 versus vehicle) decreases in IS/AR ratios were observed in ZDF rats treated with 7 and 12 mg/Kg/day telmisartan, respectively (Table 1).

### 3.2. Effects of Telmisartan on Echocardiographic Evaluations

Table 2 shows the morphometric and functional cardiac parameters following I/R. Systolic function, expressed by fractional shortening, ejection fraction, and VCF, was similar among the groups before I/R procedure. Myocardial infarction reduced the left ejection fraction (by approximately 45%); however, the LV mass and the RWT were not substantially modified. Diastolic function, expressed as isovolumetric relaxation time (IVRT), and the ratio of the maximal early diastolic peak velocity (*E*)/the late peak velocity (*A*) in milliseconds were modified by the I/R procedure. A diastolic dysfunction was induced by I/R procedure; the isovolumetric relaxation time IVRT before I/R was 28 ± 2 milliseconds in vehicle group, while being increased up to 39 ± 1 milliseconds during I/R procedure and after 2 h reperfusion. Also, the *E*/*A* ratio was diminished after I/R with values of 1.4 ± 0.1 milliseconds with respect to 2.2 ± 0.07 milliseconds before I/R.

Following telmisartan treatment, the rats showed improved cardiac parameters. The left ventricular ejection fraction LVEF after I/R was increased with respect to values observed in the vehicle group (e.g., vehicle 38 ± 2.2%; telmisartan 7 mg/kg 54 ± 2.7%). The diastolic function was also improved by telmisartan 7 mg/kg (e.g., IVRT was 30 ± 0.9 milliseconds while the *E*/*A* ratio was 1.9 ± 0.04 milliseconds) at the end of reperfusion. Myocardial performance index (MPI) measured after I/R was lower in the vehicle control group than the telmisartan 7 mg/kg treated group (e.g., 0.40 ± 0.02 versus 0.6 ± 0.01, resp.). Telmisartan 12 mg/kg produced further improvement in the cardiac parameters (Table 2).

### 3.3. Telmisartan Treatment and the Proteasome System

**Figure 1:**
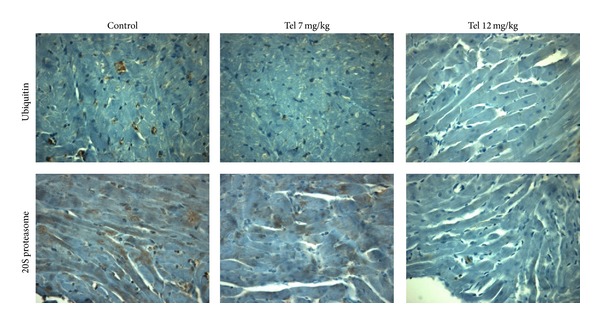
Telmisartan (tel) reduces the expression of 20S proteasome and ubiquitin in infarcted heart tissue of rats as shown by immunohistochemistry.

**Figure 2:**
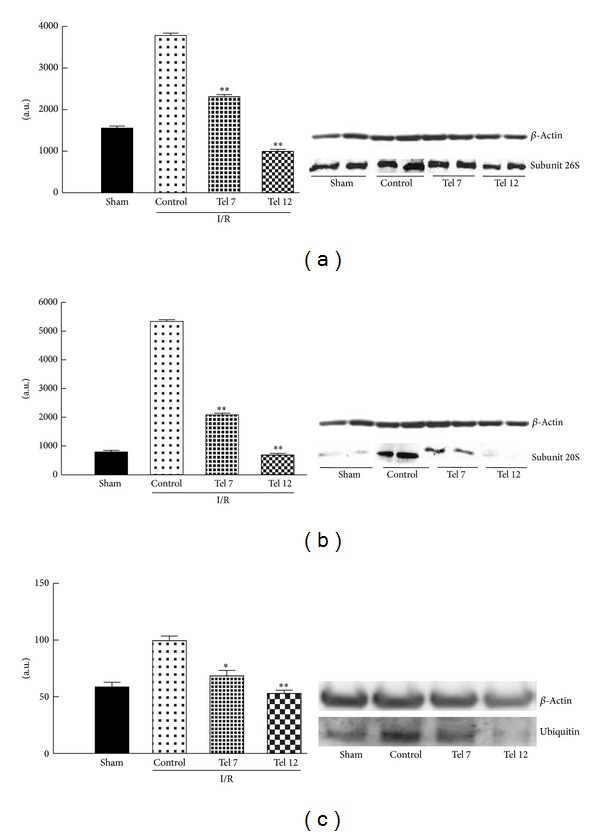
Effect of telmisartan on the expression of proteasome and ubiquitin. Telmisartan (tel) reduces in a dose-dependent fashion the expression of (a) 26S, (b) 20S proteasome subunits, and (c) ubiquitin in infarcted heart tissue of rats as shown by Western blot analysis. Values are reported as mean ± standard error. *P < 0.05 and **P < 0.01 versus control. I/R (ischemia/reperfusion); tel 7 (telmisartan 7 mg/kg); tel 12 (telmisartan 12 mg/kg).

Interestingly, following I/R procedure, there was an increase in the ubiquitin-proteasome levels within the myocardial infarcted tissue. This increase was prevented by telmisartan treatment (see Figure 1 for immunohistochemistry). Indeed, the 20S subunit was found reduced by submaximal dose of telmisartan (7 mg/kg/day for 3 weeks) of 75% and of 83% by the dose of 12 mg/kg/day, as evidenced from semiquantitative Western blot (Figure 2).

#### 3.3.1. Proteasome 20S in Macrophages Extracted from Perinfarcted Myocardial Tissue

In order to identify whether the higher proteasome levels observed in perinfarcted tissue were derived from macrophages, we repeated quantitative analyses on macrophages selectively extracted from additional 8 infarcted hearts (4 vehicles, and 4 telmisartan at 7 mg/kg). The small sample size used was not sufficient to reach an adequate statistical power for the comparisons among groups, so we can describe only the tendency of central measures for the three groups. However, we observed that the vehicle group had the highest levels of proteasome activity 20S (98.4 ± 17 pmol/mg), and the telmisartan had the lowest levels (proteasome 20S, 43.1 ± 12 pmol/mg).

#### 3.3.2. Colocalization of Proteasome 20S and Ubiquitin with Macrophages in Infarcted Tissue

**Figure 3:**
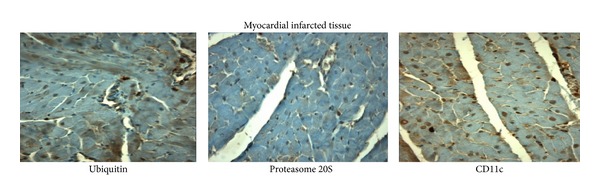
Consecutive immunostaining (X200) using serial sections of infarcted myocardial tissue demonstrated that cells positive for CD11c were also positive for proteasome 20S and ubiquitin.

Serial sections of infarcted myocardial tissue were incubated with the primary antibodies antiproteasome 20S and anti-CD11C. There was a colocalization of the proteasome and ubiquitin and M1 macrophages within the infarcted tissue, since in serial sections of infarcted tissue taken from the vehicle group the antiproteasome 20S, anti-CD11C, and antiubiquitin antibodies were present on the same cardiac cells as shown by immunohistochemistry (Figure 3).

### 3.4. Telmisartan and Cardiac Macrophage Phenotypes Polarization

**Figure 4:**
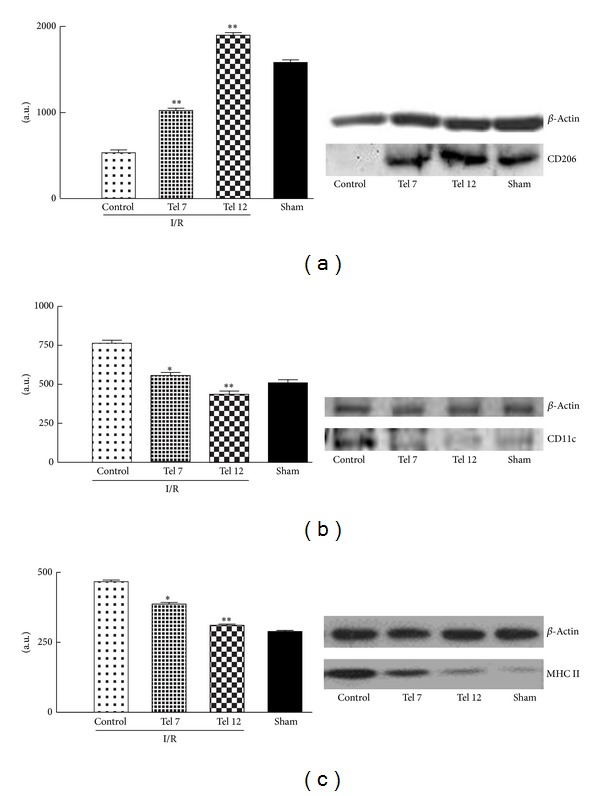
Immunoblotting for the macrophages CD206, CD11c, and MHCII in cardiac tissue of ZDF rats. (a) Expression of mannose receptor (CD206) characterizing M2 macrophage phenotype. (b, c) Expression of Integrin Alpha X/CD11c and of the major histocompatibility complex (MHC) class II characteristic M1 macrophage phenotype. Values are reported as mean ± standard error. *P < 0.05 and **P < 0.01 versus control. I/R (ischemia/reperfusion); tel 7 (telmisartan 7 mg/kg); tel 12 (telmisartan 12 mg/kg).

In conjunction with the reduction of the damage from I/R by telmisartan, we observed an increased M2 macrophage phenotype in the infarcted tissue as evidenced by the WB for the expression of the CD206 antibody. In percentage, it was ~50% for 7 mg/kg telmisartan and ~78% for telmisartan 12 mg/kg (Figure 4). We also observed a reduction in the number and levels of the macrophage M1 phenotype within the cardiac tissue of rats treated with telmisartan as evidenced by the specific CD11c binding (Figure 4).

### 3.5. Cytokines/Chemokines Profiling

**Figure 5:**
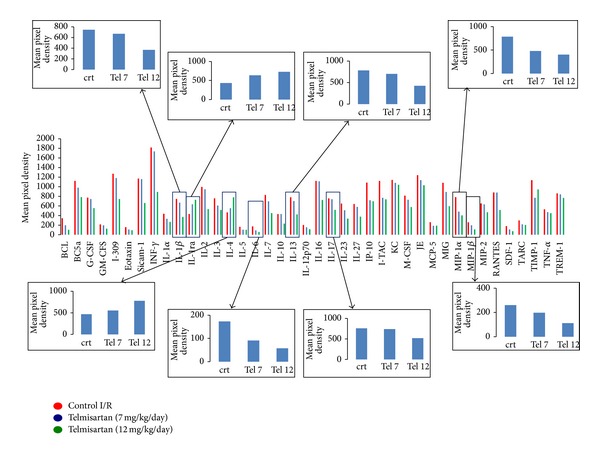
Changes caused by telmisartan (7 and 12 mg/kg) on some inflammatory and anti-inflammatory cytokines and chemokines.

Acute myocardial infarction was characterized by several cytokines and chemokines present within the tissue. The relative levels were changed following treatment of the rats with telmisartan administered prior to the induction of the I/R procedure. Among the cytokinome and chemokinome profiles within the cardiac tissue, the cytokines IL1-*β*, IL-6, IL-13, and IL-17 and the chemokines MIP-1*α* and MIP-1*β* decreased. Overall, the reduction of these cytokines was particularly evident for the dose of 12 mg/kg telmisartan (Figure 5). In contrast, we observed an increased expression of some important anti-inflammatory cytokines such as IL-4 and IL-1RA (Figure 5).

### 3.6. Cardiac Levels of MIP-1*α*

**Figure 6:**
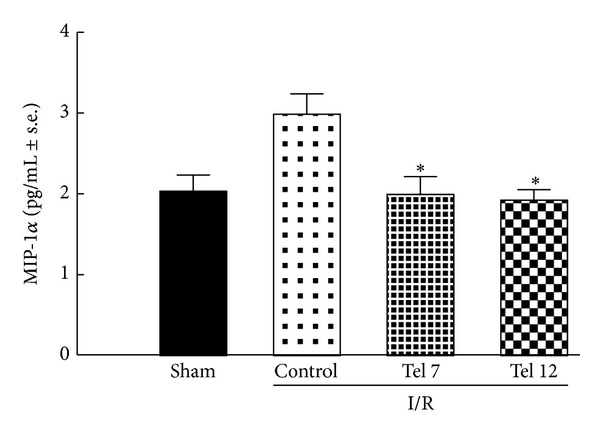
Telmisartan reduces cardiac macrophage inflammatory protein-1 alpha (MIP-1*α*) content in infarcted ZDF rats. Values are reported as means ± s.e. *P < 0.05 versus control. I/R (ischemia/reperfusion); tel 7 (telmisartan 7 mg/kg); tel 12 (telmisartan 12 mg/kg).

Among the 40 cytokines and chemokines spotted on the nitrocellulose membrane, we quantified the levels of the chemokine MIP-1*α*. The ELISA assay (R&D systems, Abingdon, UK) showed high levels of cardiac MIP-1*α* in vehicle-treated infarcted ZDF rats which decreased after treatment with 7 and 12 mg/kg/day telmisartan (Figure 6).

## 4. Discussion

In the present paper, two major mechanistic findings were evident. The first was the cardioprotection afforded by telmisartan, paralleled by the involvement of one of the most important nonlysosomal protein degradation systems, the proteasome system. The second was the discovery of great presence of M2 macrophages, a particular macrophage population associated with the resolving phase of inflammatory response within the infracted heart, following telmisartan treatment. To our knowledge, this is the first time that both the proteasome system and the macrophages M2 have been shown to be involved in the response by an angiotensin receptor blocker.

The PS is a complex protein degradation system composed of two subunits 26S and 20S, which act in contemporaneous synergism to regulate the expression of proteins and to degrade abnormal components released during severe pathologies, such as those concerning the heart [9]. The 26S is a multisubunit complex composed of a 20S proteolytic core and two 19S regulatory caps; the 20S core functions independently of ATP, whereas the 26S proteasome is an ATP-dependent system that is responsible for efficient degradation of ubiquitinated proteins. At cardiac level, the local proteasome exerts regulatory activity through several E3 ubiquitin ligases, which identify targeted proteins for ubiquitination and subsequent degradation and are involved in cardiac diseases [10]. They include atrogin-1/MAFbx (muscle atrophy F-box), MuRF (muscle RING finger), CHIP (carboxyl terminus of Hsp70-interacting protein), and MDM2 (murine double minute 2) [11]. Cardiac proteasome is also essential in activating nuclear transcription factors as, for example, the NF-*κ*B and a series of downstream events leading to the development of the inflammatory response within the myocardium [8, 12]. This group, in fact, has already shown that an inhibition of the proteasome, by using commercially specific antagonists, improves the outcome of an injury caused to the myocardium by an ischemia/reperfusion procedure through the reduction in a cascade of mediators released within the myocardium [12]. Here we show that also telmisartan does it. How telmisartan represses proteasome still warrants investigation; however, it is tempting to speculate that proteasome reduction by telmisartan may be induced by inhibition of oxidative stress and decreased ubiquitin levels as it occurs in atherosclerotic lesions [8].

The literature has demonstrated that the endogenous angiotensin system determines key events leading to early infiltration of inflammatory cells within the heart in pathologies, such as the hypertensive cardiac remodeling, the heart failure, and the AMI. This inflammatory component includes macrophages, mast cells, and T cells, which are associated with released cytokines, disruption of normal cardiac structures, and extracellular matrix deposition promoting cardiac derangement [13]. In this context we observed a high level of M1 macrophages within the infarcted tissue following myocardial infarction, as evidenced by the immunostaining performed with two specific M1 markers (e.g., Integrin Alpha X/CD11c and MHC II) on tissue taken from vehicle control rats. These macrophages are characterized by a strong propensity to antigen presentation, intra- and extracellular killing of microorganisms, and the production of high levels of cytokines (TNF-*α*, IL-12, IL-23, IL-1*β*, and IL-6) and proinflammatory mediators, such as nitric oxide and reactive oxygen intermediates [14]. On the other hand, alternative macrophage activation, also known as M2, promoted by telmisartan, is on setting. In this form of activation, macrophages are associated with resolution of inflammation and play immunoregulatory functions that contribute to the shutdown of the specific immune response previously activated and support angiogenesis and tissue remodeling, which characterize the resolution of inflammation [15, 16]. Therefore, M2 macrophages rather than M1 are crucially involved in the telmisartan cardioprotection.

According to its cardioprotection, telmisartan also modified a series of cytokines and chemokines released within the myocardium. Overall, it increases the levels of some anti-inflammatory cytokines/chemokines while decreasing the levels of proinflammatory ones. For example, telmisartan decreases the augmented levels of the cytokines IL-1*β*, IL-6, IL-13, and IL-17 and increases the levels of important anti-inflammatory cytokines such as IL-1RA and IL-4. These latter cytokines have particular protective role in immune-mediated inflammation. IL-1RA is secreted by various types of cells including immune cells, epithelial cells, and adipocytes and is a natural inhibitor of the proinflammatory effects of IL-1 alpha and IL-1 beta and modulates a variety of interleukin 1 related immune responses. IL-4 has many biological roles, including the stimulation of activated B-cells and T-cell proliferation and the differentiation of B-cells into plasma cells. It is a key regulator in humoral and adaptive immunity [17]. IL-4 induces B-cell class switching to IgE and upregulates MHC class II production. IL-4 decreases the production of Th1 cells, macrophages, IFN-gamma, and dendritic cell IL-12. The presence of IL-4 in extravascular tissues promotes alternative activation of macrophages into M2 cells and inhibits classical activation of macrophages into M1 cells. An increase in repair macrophages (M2) is coupled with secretion of IL-10 and TGF-beta that result in a diminution of pathological inflammation. On another note, IL-4 and IL-10 are massively produced by adipose tissue as defensive response to dysmetabolic stimulus [18].

Also, the chemokine macrophage inflammatory protein 1 alpha (MIP-1 alpha) is decreased by telmisartan treatment. This is a chemotactic cytokine that is now officially named CCL3 and is the major factor produced by macrophages and it is crucial for immune responses towards infection and inflammation. MIP1-alpha also induces the synthesis and release of other proinflammatory cytokines such as interleukin 1 (IL-1), IL-6, and TNF-*α* from fibroblasts and macrophages, thus amplifying the burden of negative mediators [19].

In conclusion, there is a prominent involvement of proteasome and macrophage M2 in the protection afforded by telmisartan against the acute myocardial infarction in Zucker diabetic fatty rats with metabolic syndrome.

## Figures and Tables

**Table 1:** Effects of telmisartan treatment on acute myocardial I/R injury in ZDF rats.

Drug treatment	IS (%AR)	AR (%LV)	IS (%LV)
Vehicle	68 ± 5.4	61 ± 1.8	36 ± 1.1
Tel 7 mg/kg	48 ± 4.7*	58 ± 1.5	28 ± 2.9*
Tel 12 mg/kg	30 ± 3.4**	59 ± 1.3	20 ± 1.8**

Tel = telmisartan; I/R = ischemia/reperfusion; IS = infarct size; AR = area at risk; LV = left ventricle. *P < 0.05 and **P < 0.01 versus vehicle.

**Table 2:** Cardiac parameters evaluated by echocardiography in ZDF rats before and after acute myocardial infarct.

Variable	Before I/R			After 2 h reperfusion		
	Vehicle	Tel 7	Tel 12	Vehicle	Tel 7	Tel 12
Morphometric						
LV mass (g)	0.9 ± 0.09	0.8 ± 0.07	0.78 ± 0.06	0.8 ± 0.05	0.9 ± 0.05	0.88 ± 0.04
RWT	0.4 ± 0.05	0.35 ± 0.06	0.32 ± 0.04	0.5 ± 0.04	0.4 ± 0.03	0.39 ± 0.04

Systolic function						
LVFS (%)	48 ± 1.3	50 ± 1.5	51 ± 1.4	40 ± 1.7	49 ± 2*	52 ± 1.9**
LVEF (%)	53 ± 3	62 ± 3.1*	65 ± 2.9**	38 ± 2.2	56 ± 2.7**	60 ± 2.5**
VCF (circ/sec)	0.006 ± 0.0002	0.0057 ± 0.0001	0.0056 ± 0.0003	0.004 ± 0.0003	0.007 ± 0.0003	0.008 ± 0.0004

Diastolic function						
IVRT (ms)	28 ± 2	29 ± 3	29.7 ± 4	39 ± 1	30 ± 0.9	28 ± 0.7
E/A ratio (ms)	2.2 ± 0.07	2.5 ± 0.07**	2.6 ± 0.06	1.4 ± 0.1	1.9 ± 0.04**	2.1 ± 0.03**

Global index						
MPI	0.7 ± 0.01	0.8 ± 0.02**	0.82 ± 0.04**	0.40 ± 0.02	0.6 ± 0.01**	0.65 ± 0.03**

Tel 7 = telmisartan (7 mg/kg); Tel 12 = telmisartan (12 mg/kg); LV = left ventricular mass and relative wall thickness (RWT); EF = ejection fraction; FS = fractional shortening and velocity of circumferential fiber shortening (VCF); IVRT = absolute left ventricular isovolumetric relaxation time; E/A ratio = ratio of maximal early diastolic peak velocity/late peak velocity of mitral flow. MPI = myocardial performance index; ms = milliseconds. *P < 0.05 and **P < 0.01 versus vehicle.
